# Integrated profiling of microRNA expression in membranous nephropathy using high-throughput sequencing technology

**DOI:** 10.3892/ijmm.2013.1554

**Published:** 2013-11-12

**Authors:** WENBIAO CHEN, XIAOCONG LIN, JIANRONG HUANG, KUIBI TAN, YUYU CHEN, WUJIAN PENG, WUXIAN LI, YONG DAI

**Affiliations:** 1 Second Clinical Medical College of Jinan University, Shenzhen People’s Hospital, Shenzhen, Guangdong, P.R. China; 2 Institute of Biochemistry and Molecular Biology, Guangdong Medical College, Zhanjiang, Guangdong, P.R. China; 3 Key Laboratory of Laboratory Medical Diagnostics of the Ministry of Education, Faculty of Laboratory Medicine, Chongqiong Medical University, Chongqing, Sichuan, P.R. China

**Keywords:** membranous nephropathy, microRNA, novel microRNA, high-throughput sequencing, microRNA base edit, expression and distribution

## Abstract

The present study analyzed microRNA (miRNA) expression profiles in peripheral blood lymphocyte cells (PBLCs) from patients with membranous nephropathy (MN) and normal controls (NC), in an effort to improve the understanding of the pathogenesis of MN. High-throughput sequencing was performed on 30 MN patients and 30 healthy individuals (NC group). Known and novel miRNAs were analyzed and the results were confirmed by quantitative reverse transcription PCR (qRT-PCR). In total, 326 miRNAs showed a significant difference in expression between the MN and NC groups. This included 286 downregulated miRNAs and 40 upregulated miRNAs. In addition, there were 6 novel miRNAs that presented differential levels of expression between the MN and NC groups. The miRNAs were mapped to the genome, using a short oligonucleotide alignment program (SOAP), to analyze their expression and distribution. Twenty-five percent of the unique miRNAs in the MN group and 52.1% in the NC group were mapped to the genome. One hundred and eight mismatches were identified. Seventy-seven mismatches were detected in a higher proportion of the MN samples, compared with the NC samples. Twenty-five mismatches were detected in a higher proportion of the NC samples than the MN samples. Differential miRNA expression was also detected between 10 randomly selected pair groups, as depicted in a cluster analysis diagram. These data indicate that differential miRNA expression may be involved in the pathogenesis of MN. In addition, the discrepancies between the MN and NC groups, in the mismatched miRNAs that were mapped to the genome, strongly suggest that miRNAs play an important role in the pathogenesis of human disorders. miRNAs may provide a potential breakthrough in the research of MN and may provide a novel biomarker for the diagnosis and treatment of the disease.

## Introduction

Membranous nephropathy (MN) is a renal disease that is histologically characterized by the uniform thickening of the glomerular capillary wall. This is caused by subepithelial deposits of immune complexes, which appear as granular deposits of immunoglobulin G when visualized using immunofluorescence, and as an electron-dense deposit when observed under an electron microscope. MN is a frequent cause of nephrotic syndrome in adults and can eventually progress to end-stage renal failure in many patients (1–3). The MN disease site is the glomerular visceral epithelial cell, or podocyte, which is a highly specialized and terminally differentiated cell that is found on the outside of the glomerular basement membrane (4). The disease, similar to most immune glomerular diseases, manifests itself as an immune response to the self-antigens expressed on the podocyte cell membrane (5). There is no specific treatment for MN. Diuretics and angiotensin-converting enzyme inhibitors have limited effects, whilst immunosuppressants cause side-effects and their use in treatment is controversial (6).

Currently, the diagnosis of kidney disease depends mainly on renal biopsy and immunofluorescence (7). There is an urgent need for a validated biomarker that is easy to measure and able to accurately predict long-term outcomes, to aid both in the diagnosis and treatment of the disease (8). The clinical management of patients with MN may improve with the better understanding of the underlying mechanisms of the disease and the availability of biomarkers.

microRNAs (miRNAs) regulate gene expression and have been found to modulate crucial biological processes, including differentiation, proliferation and apoptosis (9). They function through various mechanisms, such as targeted miRNA degradation and translational inhibition (10,11). Hundreds of miRNAs have been identified, using molecular cloning and bioinformatics prediction strategies, in worms, flies, fish, frogs, mammals and flowering plants (12). Several studies have indicated a possible link between miRNAs and kidney disease (13–15). Although the expression results of the various array analyses are inconsistent, the data indicate that the dysregulation of miRNAs may play a pivotal role in the pathogenesis of kidney disease. However, there have been few studies on the association between miRNAs and MN. In this study, we analyzed the pathogenesis of MN at the miRNA level. Next generation high-throughput sequencing is an effective technique that enables miRNA profiling at unprecedented quantitative and qualitative levels (16). The data presented in this study may enhance our knowledge of miRNA expression profiles in patients with MN, and may provided insight into the pathogenesis, diagnosis and treatment of MN.

## Patients and methods

### Patient samples

Peripheral blood samples were collected from 30 patients at the Second Clinical Medical College, Jinan University, Shenzhen People’s Hospital (Shenzhen, China) between 2011 and 2012. The diagnosis of MN was confirmed by renal biopsy and immunofluorescence. Thirty specimens for the control group were obtained from individuals who underwent annual body check-ups and were confirmed as healthy in 2012, at Shenzhen People’s Hospital.

Prior written informed consent was obtained from all patients. The project was approved by the Shenzhen People’s Hospital Ethics Committee. This study was performed in accordance with the guidelines of Jinan University, which abides by the Helsinki Declaration on ethical principles for medical research involving human subjects.

### Sample processing

Samples were collected in EDTA tubes and then transferred to depletion filters to separate the lymphocytes, in accordance with the LeukoLock Total RNA isolation system protocol (Ambion, Austin, TX, USA). Total RNA was extracted from the cells using the miRNeasy Mini kit (Qiagen, Hilden, Germany), in accordance with the manufacturer’s instructions. The integrity of the RNA and the presence of miRNAs were assessed by micro-capillary electrophoresis, using an RNA 6000 kit and small RNA kit (Agilent Technologies Inc., Santa Clara, CA, USA), respectively. The concentration and quality of the RNA were assessed by absorbance spectrometry on a NanoDrop 2000 spectrophotometer (Thermo, Waltham, MA, USA).

### miRNA microarray

miRNA microarrays, composed of 455 human, 236 rat and 344 mouse miRNAs, were used to detect all human, rat, mouse and other miRNAs in the Sanger miRNA database (release 8.1). This study used miRNA power labeling (Exiqon, Vedbaek, Denmark) to 3′- or 5′-end label 0.5 μl of a sample or a human universal reference total RNA, with the cy3-like HY3 or cy5-like HY5 dye, respectively. Locked nucleic acids (LNAs) were used, as their superior sensitivity over conventional DNA-based miRNA arrays can discriminate between closely related miRNA family members.

### miRNA microarray analysis

**Figure 1:**
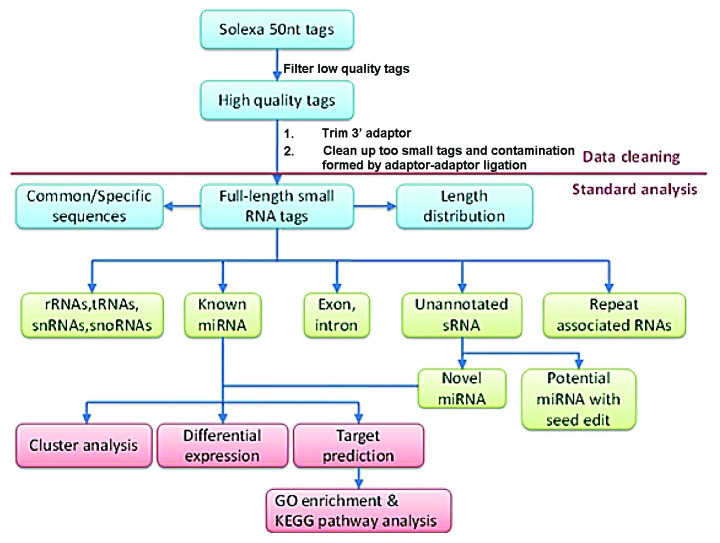
MicroRNA (miRNA) microarray analysis process. The data analysis process used 50 nucleotide tags that were produced by high-throughput sequencing. The low quality reads were filtered out in accordance with the base quality value. The adaptor sequence was trimmed at the 3′-terminus. The 5′ adaptor contaminants that were formed by ligation were removed. The clean, high-quality reads were used for standard analysis. Cluster analysis contained the data on small RNA expression and distribution in each genome. Differential expression included miRNA and novel miRNA expression in the membranous nephropathy (MN) and normal control (NC) groups. Target prediction was analyzed using the summary of known miRNA alignments and miRNA base edits. rRNA, ribosomal RNA; tRNA, transfer RNA; snRNA, small nuclear RNA; snoRNA, small nucleolar RNA; sRNA, small RNA.

To create an MN library and normal control (NC) library, total miRNAs from each subject underwent miRNA library construction and sequencing. The process is illustrated in Fig. 1. The 50 nucleotide sequence tags used for the high-throughput sequencing were obtained through data cleansing to remove low quality tags and several types of contaminants. The length distribution of the clean tags was summarized and used in a standard bioinformatics analysis. The clean tags were annotated and placed into different categories to predict novel miRNAs and edit known miRNAs. The tags that could not be annotated were placed in a separate category.

### Statistical analysis

Statistical analyses were performed after the libraries were established. The miRNAs were mapped to the genome and a summary was produced for the known miRNA alignments. Cluster analysis, differential expression and base edit analysis were performed for the miRNAs.

### Quantitative reverse transcription PCR (qRT-PCR) verification of miRNA results

qRT-PCR was performed to verify the results of the deep sequencing analysis for 5 differentially expressed miRNAs. Total RNA (2 μg) was reverse transcribed into cDNA using a reverse transcription kit, in accordance with the manufacturer’s instructions (Promega, Madison, WI, USA). The cycle parameters for the PCR reaction were as follows: 95°C for 5 min followed by 40 cycles of a denaturing step at 95°C for 10 sec and an extension step at 60°C for 60 sec. All reactions were run in triplicate. The relative quantification 2^−ΔΔCt^ method was used to determine the changes in expression levels between the MN and NC groups. The miRNA expression levels were normalized to the reference RNA, RUN6. The −ΔΔCt values were calculated using the following formula: −ΔΔCt = −Ct_(MN − NC)_, where Ct is the cycle threshold provided by the Rotor-Gene 6000 Series Software 1.7 (Qiagen).

## Results

### Small RNA expression and distribution in each genome

**Figure 2:**
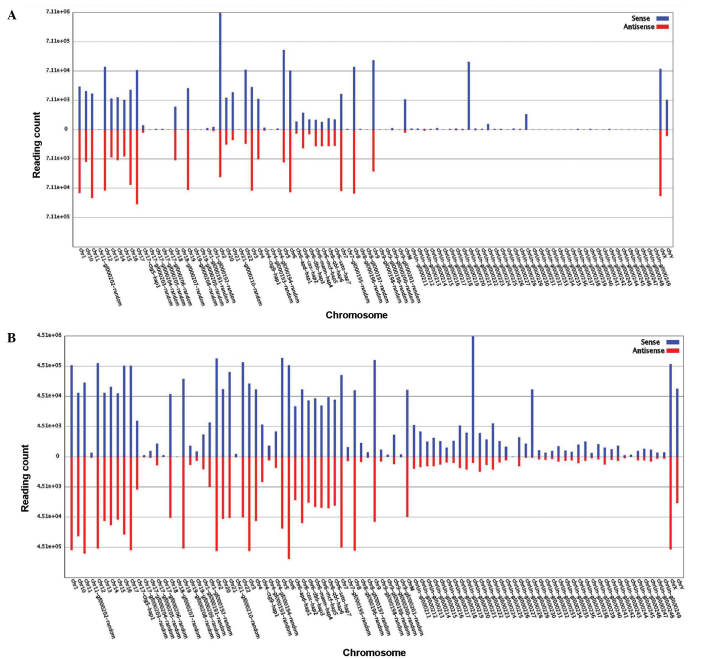
Number of small RNA tags that were located on each chromosome in the (A) membranous nephropathy (MN) and (B) normal control (NC) groups. The area above 0 is the number of small RNAs on the sense strand of the chromosome, shown in blue. The area below 0 is the number of small RNAs on the antisense strand of the chromosome, shown in red.

High-throughput sequencing produced 11,436,003 and 18,696,751 high quality sequence reads from the MN and NC groups, respectively. Following the removal of the contaminated reads, the total number of clean, small RNA reads was 11,109,127 and 16,001,191, in the MN and NC groups, respectively. The number of unique small RNAs was 154,580 in the MN group and 1,034,806 in the NC group. The small RNA tags were mapped to the genome using the short oligonucleotide alignment program (SOAP) program, as previously described (17), to analyze their expression and distribution. The results are presented in Table I. The percentage of unique small RNAs mapped to the genome in the NC group was 52.10%, which was greater than the 25% observed for the MN group. By contrast, the MN percentage for the total small RNAs was 85.00%, whilst for the NC group the value was 83.10%. Fig. 2 shows the number of small RNA tags that were located on each chromosome and the comparison between the MN and NC groups.

### Summary of known miRNA alignments

**Figure 3:**
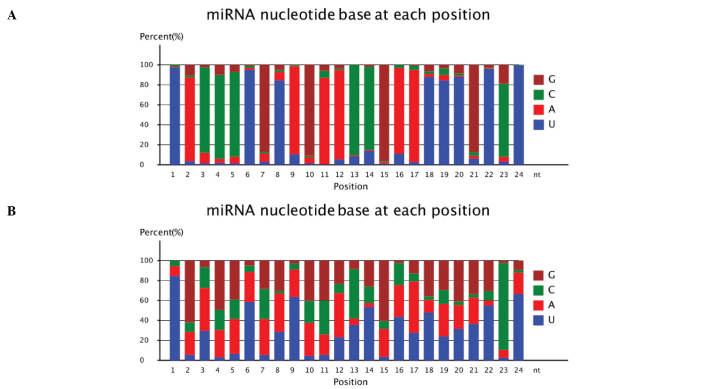
MicroRNA (miRNA) nucleotide base (A/U/C/G) at each position in (A) the membranous nephropathy (MN) and (B) normal control (NC) groups. The percentge of different nucleotide bases at each position. Brown represents G, green represents C, red represents A, blue represents U. A, adenine, U, uracil, C, cytosine, D, guanine.

We aligned the small RNAs to miRNA precursors in the corresponding species to obtain the miRNA count. This also allowed the identification of bases at each position within the miRNAs. The results are shown in Table II. The percentage occurence of each base (A/U/C/G; A, adenine, U, uracil, C, cytosine, D, guanine) at each position in the small RNAs was calculated. A large variation was observed between the MN and NC groups. For example, 83.99% of the bases in the second position were A in the MN group. Only 22.99% of the bases at this position were A in the NC group. Significant differences were also observed at the 9th base (10.10% U in the MN group, 63.04% U in the NC group) and 14th base (83.09% C in the MN group, 16.62% C in the NC group). However, some similarities were observed between the two groups at base positions 1, 6, 10, 12, 13, 15, 17, 18, 22, 23 and 24. At these positions, the most common base was the same in each group. The outcomes are depicted in Fig. 3.

### Differential expression of miRNAs in the MN and NC groups

Four procedures were completed to compare the miRNA expression between the MN and NC libraries: i) relative expression analysis was used to normalize the data against the number of miRNAs and the total number of small RNA reads. This was used to define the expression preferences of individual miRNAs, between the two libraries. ii) The outcome of the relative expression analysis was multiplied by a constant, set at 1×10^6^: Normalized expression = (actual miRNA count/total count of clean reads) ×10^6^. iii) The fold change and P-values were calculated from the normalized expression data: fold change = log_2_ (MN/NC), P(y/x) = (N_2_/N_1_)^y^ × (x+y)!/x!y!(1+N_2_/N_1_)^(x+y+1)^ (x and y indicate the number of reads of a miRNA in the NC and MN libraries, respectively. N_1_ and N_2_ represent the total number of clean reads in the NC and MN libraries, respectively). iv) The fold change and Audic-Claverie method (18) were used to define the differential expression of miRNAs between the two groups. Fold changes (log_2_ MN/NC) with P≤0.01 were considered to indicate a statistically significant result.

**Figure 4:**
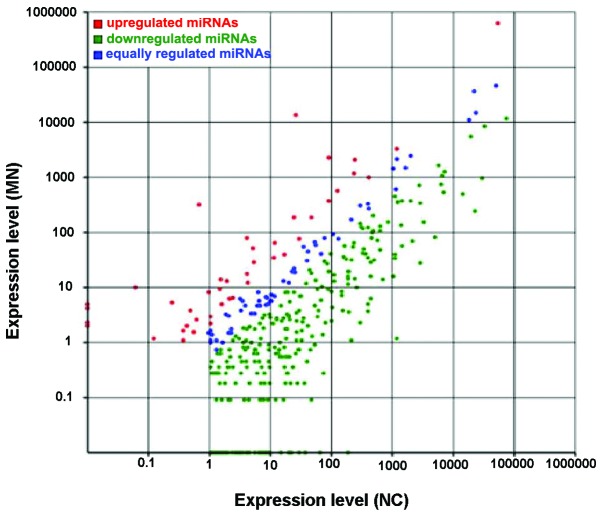
Scatter plot of differential expression of microRNAs (miRNAs) between the membranous nephropathy (MN) and normal control (NC) libraries. Red indicates upregulated miRNAs [log_2_ (MN/NC) >0], blue indicates miRNAs with no change in expression between MN and NC libraries (no meaning in the research), green indicates downregulated miRNAs [log_2_ (MN/NC) <0].

Differential expression between the MN and NC libraries was found in 326 miRNAs. These consisted of 286 miRNAs that were downregulated and 40 miRNAs that were upregulated (Fig. 4). The fold change value for the downregulated miRNAs ranged from −14.23 (hsa-miR-217) to −1.03 (hsa-miR-589-5p). The greatest fold change in the upregulated miRNAs was 8.97 (hsa-miR-486-5p). No upregulated miRNA exceeded a fold change of 10. The 20 upregulated and downregulated miRNAs with the highest fold changes in expression are listed in Table III.

### Differential expression of novel miRNAs in the MN and NC groups

There were 15 novel miRNAs in the MN group and 22 novel miRNAs in the NC group. Only 6 of these showed a significantly different level of expression between the MN and NC groups. The novel-miR-82, novel-miR-98, novel-miR-89 and novel-miR-84 miRNAs were downregulated, whilst the novel-miR-152 and novel-miR-15 miRNAs were upregulated (Table IV). The novel-miR-15 fold change of 9.85 was the largest fold change in the upregulated group. The novel-miR-84 miRNA with the fold change of −7.45 was the largest in the downregulated group.

### Cluster analysis of miRNAs

**Figure 5:**
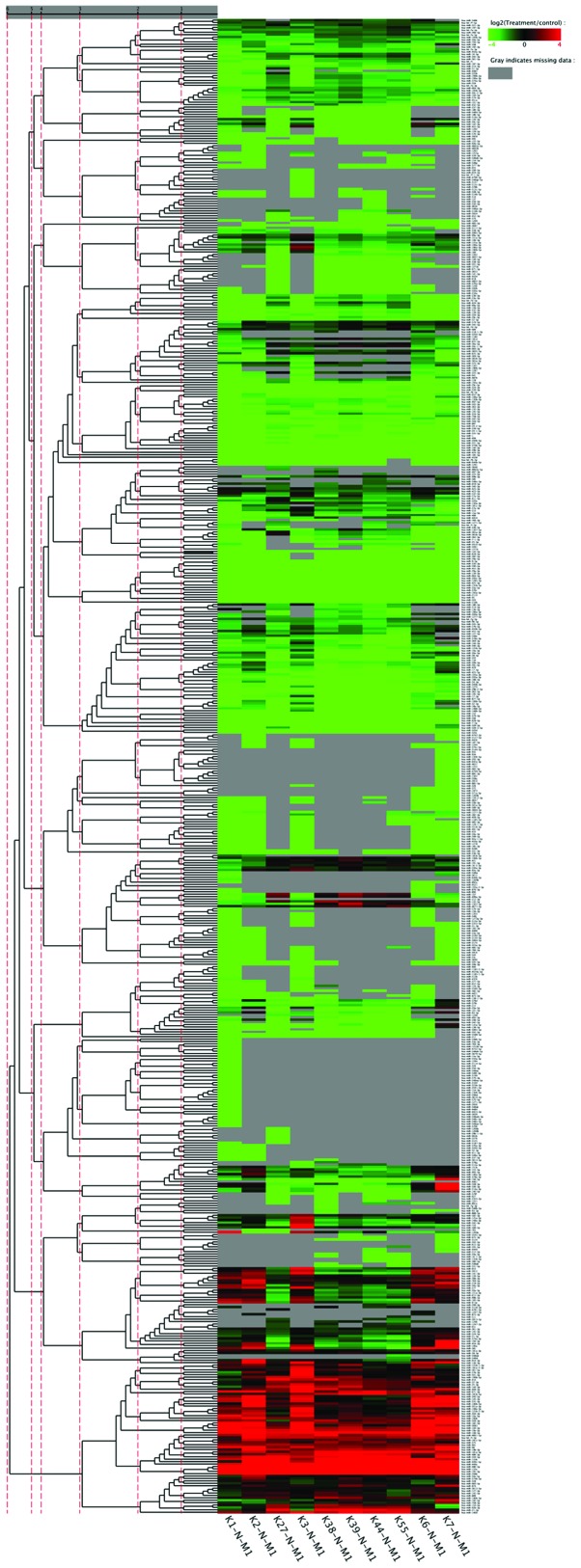
Heat map cluster analysis of microRNAs (miRNAs). Each row in the figure represents one miRNA. Each column represents one sample pair [one sample from patients with membranous nephropathy (MN) and one sample from normal controls (NC)]. Each cell represents the differential expression of an miRNA in a sample pair.

From the 30 specimens in the NC group, 10 were randomly selected and labeled as K7-N, K6-N, K55-N, K44-N, K39-N, K38-N, K3-N, K27-N, K2-N and K1-N and were compared with the MN specimens in a heat map (Fig. 5). The samples were clustered in accordance with their similarities in expression patterns, i.e., fold change and P-value. Red indicated that the miRNA had a higher expression level in the MN specimens, whilst green indicated that the miRNA had a higher expression level in the 10 specimens from the NC group. Gray indicated that the miRNA was not expressed in at least one sample.

### miRNA base edits

Nucleotide base positions 2–8 of a mature miRNA are highly conserved and are known as the seed region. The target of the miRNA may be dependent on this region. In our analysis, seed region base changes were detected by aligning unannotated small RNAs with mature miRNAs from the miRBase 18 database (http://www.mirbase.org/). Mismatches in the alignment were assumed to be associated with the mechanism of the disease. In this study, 108 miRNAs, which were common between the MN and NC groups were analyzed for base edits. The proportion of miRNAs with base edits was used to obtain the ratio of base edits between the two groups (Table V). There were 77 miRNAs in which there were more base edits in the MN group than in the NC group (MN/NC >1), 6 miRNAs that were equivalent between the two groups (MN/NC = 1) and 25 miRNAs in which there were more base edits in the NC group than in the MN group (MN/NC <1). Generally, miRNA base edits occurred in the MN group more often than in the NC group, which indicated a link with the disease.

### Validation of miRNA expression by qRT-PCR

The expression levels of 5 randomly selected miRNAs: hsa-miR-7-5p, hsa-miR-615-3p, hsa-miR-577, hsa-miR-98 and hsa-miR-375, were compared. The hsa-miR-98 and hsa-miR-375 miRNAs were upregulated, whilst hsa-miR-7-5p, hsa-miR-615-3p and hsa-miR-577 were downregulated. The qRT-PCR data were obtained using the 2^−ΔΔCt^ method and normalized using RNA RUN6 as a reference. The log_2_ (MN/NC) value was compared with the fold change value (Table VI). The log_2_ (MN/NC) values of hsa-miR-7-5p, hsa-miR-615-3p and hsa-miR-577 were −5.06, −2.40 and −1.12, respectively. These data confirmed that these miRNAs were downregulated. The log_2_ (MN/NC) values of hsa-miR-98 and hsa-miR-375 were 4.28 and 2.63, respectively, again confirming the previous data, indicating that they were upregulated.

## Discussion

The high through-put sequencing used in this study is suitable for the analysis of small RNA molecules as it is able to decrease the loss of nucleotides in the reads, caused by secondary structure. The technology is also ideal as it does not require a large sample quantity (19). Such an analysis can obtain millions of small RNA sequence tags in one run and can identify the differential expression of small RNAs between two samples (20). We performed high through-put sequencing on a large number of peripheral blood lymphocytes from individuals separated into the MN and NC groups. The aim was to identify dysregulated miRNAs that may serve as reliable diagnostic markers and potential therapeutic targets. The data confirmed that dysregulated miRNAs may play an important role in the pathogenesis of nephropathy, which is consistent with previous studies (21–23).

We determined the unique and total number of small RNAs in the MN and NC groups, and positioned the small RNAs within the genome. Expression analysis and distribution of the small RNAs was also performed. The number of total and unique small RNAs was greater in the NC group than in the MN group. The miRBase 18 database (http://www.mirbase.org/) provides a range of data to facilitate studies of miRNA genomics. This has been used previously to map all miRNAs to their genomic coordinates (24), allowing a network of genome-wide miRNA expression to be produced (25,26). Analysis of the properties of miRNA targets is a promising approach to the prediction of miRNA function. If the targets of specific miRNAs are enriched with genes associated with a particular biological process, it is reasonable to infer that the miRNA is also involved in the same process (27). The function of miRNAs was not predicted in this study; however, a statistical analysis was performed, revealing a discrepancy between a diseased and a normal group of specimens, regardless of the quantity and distribution of miRNAs. This provided strong evidence for the function of miRNAs in the pathogenesis of disease.

Our data demonstrated the bias in miRNA nucleotides at each base position. We found that U was the dominant nucleotide in miRNAs. This was particularly noticeable at positions 1, 6, 8, 18, 19, 20, 22 and 24 in the MN group, and at positions 1, 6, 9, 14, 22 and 24 in the NC group. These results are consistent with those of Zhang *et al*(28), who demonstrated that the distribution of nucleotides indicated an important role for U at the boundaries of the seed region and termini. In addition, there was a large discrepancy in the proportion of the 4 bases at each position. For example, U accounted for 96.61 and 84.27% of bases at the 1st position in the MN and NC groups, respectively. The A base accounted for 92.45 and 51.50% of bases at the 17th position in the MN and NC groups, respectively. Genes with a higher level of expression showed stronger signals, which indicated that these nucleotides were responsible for the regulation of translation initiation. The diversity of nucleotide sequences surrounding the initiation codon has been explained by differences in relative contributions from two distinct patterns (29), and preferred nucleotide sequences varied between different eukaryotic species (30). We speculate that the reasons for the different nucleotide bias, between the MN and NC groups, resulted from the individual differences in evolution or the role of miRNAs. This requires further research.

In the present study, the results of the miRNA differential expression analysis were unexpected as there were more downregulated (n=286) miRNAs than upregulated (n=40) miRNAs. By contrast, there are several reports on miRNA pathogenesis in nephropathy that have reported more upregulated miRNAs, compared with downregulated miRNAs (13,31). This is also the case for diseases, such as congenital disorders (32), cancer (33) and immunological diseases (34). However, certain studies have reported an increase in downregulated miRNAs, compared with upregulated miRNAs; however, the difference in numbers is small. Chen *et al* reported more downregulated (n=41) miRNAs than upregulated (n=33) ones in urothelial cell carcinoma (35). Osanto *et al* reported more downregulated (n=41) miRNAs than upregulated (n=29) ones in clear cell renal cell carcinoma (36). miRNAs that are more abundant in the kidneys, compared with other organs, include miR-192, miR-194, miR-204, miR-215 and miR-216 (37). The miRNA-30 family (hsa-miR-30e-5p, hsa-miR-30e-3p, hsa-miR-30d-5p, hsa-miR-30c-5p, hsa-miR-30c-2-3p, hsa-miR-3b-5p, hsa-miR-30b-3p, hsa-miR-3a-5p and hsa-miR-30a-3p) and the miR-133 family (hsa-miR-133b and hsa-miR-133a) have been linked to the connective tissue growth factor (CTGF), which is a key molecule in the process of fibrosis (38). It is also a key molecule in the process of nephropathy (39). Our study demonstrated that the miRNA-30 family was downregulated and that the miR-133 family was upregulated. The loss of miR-23b, miR-24 and miR-26a resulted in the rapid progression of marked glomerular and tubular injury. Their existence has been shown to be critical in maintaining glomerular filtration (40). These miRNAs were also downregulated in our study. We deduced that the key involvement of miRNAs in the pathogenesis of MN was an outcome of downregulation. This could explain why the downregulation of miRNAs was more common than the upregulation. However, our inference requires a more in-depth study.

We were also able to predict novel miRNAs, with 6 that showed a significant difference in expression between the MN and NC groups. Four of the 6 were downregulated and 2 were upregulated. The outcome was in agreement with the higher numbers of miRNAs that were downregulated, compared with the number that were upregulated, as described earlier in this study. Certain studies have reported that the read number for most novel miRNAs is much lower than that for the conserved miRNAs, which indicates that non-conserved miRNAs are usually expressed at a lower level (35,41). Despite the limited number of novel miRNAs in this study, we found that the fold change was relatively large, with values >6 or <−6. This indicated that the novel miRNAs may have functional relevance in the pathogenesis of MN. The identification of novel miRNA genes is important as it may reveal putative genes that exert a regulatory effect on different types of cancer (42). Dhahbi *et al*(43) found 20 novel miRNAs that were differentially expressed between young and senescent fibroblasts. Three novel miRNAs have been shown to exhibit relative sequence counts of >10 and are likely to be involved in the development of prostate cancer (41). These studies demonstrated that novel miRNAs have are closely associated with the occurrence of disease. The targets and functions of novel miRNAs, which had not previously been investigated in MN, have yet to be determined.

The nucleotides at positions 2–8 of mature miRNAs are known to be highly conserved. The targets of miRNAs may be altered by a change in the nucleotides in this region (44). We calculated the percentage of base edits and compared the percentages between the MN and NC groups. A discrepancy was observed, which indicated that this region may be implicated in the pathogenesis of the disease. Blow *et al* found that 6 of 99 surveyed pre-miRNAs were edited in at least 1 of 10 human tissues (45). The hsa-miR-1269b and hsa-miR-1034-3p miRNAs had the largest percentage (100%) of edited bases in the NC group. There were 9 miRNAs in the MN group where the percentage of edited bases reached 100%. There was a similar base edit percentage in both the MN and NC groups. For example, hsa-let-7a-5p, 85.52% in the MN group and 64.99% in the NC group; hsa-miR-1304-4p, 100% in the MN group and 100% in the NC group; hsa-miR-130-3p, 5.54% in the MN group and 2.10% in the NC group. We identified that the number of miRNAs with a ratio (MN)/ratio(NC) >1 was 77, whilst those with a ratio (MN)/ratio(NC) <1 was 25. Kawahara *et al* provided the first evidence that edited miRNAs have a biological significance *in vivo*(46). We speculated that miRNA base edits may be involved in the pathogenesis of MN and may provide an innovative method for investigating the mechanisms responsible for the development of MN.

This study is one of the few that have profiled the expression patterns of miRNAs on a genome-wide scale in patients with MN. Our results demonstrated the differential expression of miRNAs and a discrepancy in nucleotide bias between normal and diseased individuals. Base edits showed a clear difference between the two groups, which strongly indicated that dysregulated miRNAs may present an area of research into the pathogenesis of MN. Our study also indicated that miRNAs can serve as a potential clinical target to diagnose and treat MN patients in the future. Whilst the results indicated the significant potential and benefit of miRNAs, further studies are required to provide further insight into the molecular functions of miRNAs. Our data may be used as basic research to support novel methods for the investigation, diagnosis and treatment of MN. We anticipate that miRNA-based genetic therapies will be developed to replace traditional therapies for the future benefit of patients.

## Figures and Tables

**Table I:** Mapping of unique small RNAs and total small RNAs to the genome.

	Unique sRNAs^a^		Total sRNAs^b^	
		
	MN	NC	MN	NC
Total sRNAs	154,580	1,034,806	11,109,127	16,001,191
Mapping to the genome	38,577	539,279	9,445,450	13,301,445
Percentage (%)^c^	25.00	52.10	85.00	83.10

a Number and percentage of unique small RNAs (sRNAs) that were mapped to the genome.

b Number of total sRNAs that were mapped to the genome.

c The outcome, calculated by sRNAs mapped to the genome/total sRNAs.

MN, membranous nephropathy; NC, normal controls.

**Table II:** Percentage occurence of each base (A/U/C/G) at each position in the sRNAs in the MN and NC group.

A, MN group				

Position on sRNA	A (%)	U (%)	C (%)	G (%)
1	0.90	96.61	2.40	0.09
2	83.99	3.11	2.01	10.89
3	10.41	1.42	85.66	2.51
4	3.96	1.95	83.73	10.36
5	6.30	1.28	85.09	7.33
6	1.70	94.87	2.23	1.19
7	7.77	2.78	1.94	87.51
8	8.79	84.02	1.92	5.27
9	88.05	10.10	0.93	0.92
10	5.85	1.18	1.68	91.29
11	86.34	0.46	6.70	6.49
12	88.81	5.31	1.45	4.43
13	1.27	8.18	90.15	0.41
14	0.92	13.78	83.09	2.20
15	1.62	0.75	0.86	96.78
16	86.03	10.53	3.04	0.40
17	92.45	2.44	3.83	1.28
18	2.82	87.87	2.79	6.51
19	5.16	84.35	6.62	3.87
20	1.09	87.95	2.34	8.63
21	3.15	5.76	3.20	87.90
22	0.78	95.56	0.24	3.43
23	5.13	2.72	72.78	19.37
24	0.00	99.37	0.51	0.12

B, NC group				

Position on sRNA	A (%)	U (%)	C (%)	G (%)

1	10.01	84.27	5.62	0.10
2	22.99	5.07	9.87	62.07
3	42.97	29.38	20.91	6.74
4	27.54	2.84	19.77	49.84
5	34.92	6.37	19.69	39.02
6	29.87	58.16	6.84	5.12
7	35.85	5.20	29.98	28.96
8	38.34	28.22	2.58	30.85
9	27.48	63.04	6.42	3.05
10	32.86	4.33	22.10	40.71
11	20.43	5.49	34.07	40.01
12	44.81	22.61	8.66	23.93
13	6.28	35.38	49.68	8.66
14	3.93	53.36	16.62	26.09
15	27.70	3.42	8.18	60.71
16	31.53	43.49	22.36	2.62
17	51.50	27.04	8.13	13.33
18	12.07	47.97	3.64	36.32
19	32.18	23.90	13.92	29.99
20	24.02	31.37	3.44	41.18
21	26.34	36.24	3.54	33.87
22	4.88	54.75	9.62	30.76
23	8.07	2.10	86.98	2.84
24	21.44	66.17	2.77	9.62

The percentage of a base at a particular position is shown. A, adenine; U, uracil; C, cytosine; D, guanine. MN, membranous nephropathy; NC, normal controls.

**Table III:** The top 20 upregulated and downregulated miRNAs.

miRNA name	NC^a^	MN^b^	Fold change (log_2_)^c^	P-value
Downregulated miRNAs				
hsa-miR-217	191.8607	0.01	−14.22777294	0
hsa-miR-216a	66.6825	0.01	−12.70309232	5.5967E-245
hsa-miR-216b	38.1222	0.01	−11.89641592	2.4708E-140
hsa-miR-95	29.9978	0.01	−11.5506409	1.4472E-110
hsa-miR-671-5p	22.1858	0.01	−11.11542074	6.07198E-82
hsa-miR-3653	20.311	0.01	−10.98804541	4.49572E-75
hsa-miR-1285-3p	14.8739	0.01	−10.53856732	3.75197E-55
hsa-miR-200c-3p	1181.5995	1.1702	−9.97977026	0
hsa-miR-29a-5p	9.9993	0.01	−9.9656833	2.72194E-37
hsa-miR-627	9.7493	0.01	−9.92915478	2.24289E-36
hsa-miR-425-3p	9.4368	0.01	−9.88215398	3.13127E-35
hsa-miR-503	8.7493	0.01	−9.77302376	1.03401E-32
hsa-miR-193a-5p	8.3744	0.01	−9.70984202	2.44579E-31
hsa-miR-382-5p	7.6869	0.01	−9.58625814	8.07652E-29
hsa-miR-135b-5p	7.1245	0.01	−9.47664493	9.29106E-27
hsa-miR-4802-3p	6.812	0.01	−9.41193458	1.29711E-25
hsa-miR-1246	6.812	0.01	−9.41193458	1.29711E-25
hsa-miR-500a-5p	6.6245	0.01	−9.37166775	6.30846E-25
hsa-miR-500b	6.562	0.01	−9.35799181	1.06882E-24
hsa-miR-5588-5p	5.6246	0.01	−9.1356067	2.90831E-21
Upregulated miRNAs				
hsa-miR-486-5p	26.5605	13344.793	8.97277891	0
hsa-miR-208b	0.01	4.8609	8.92507964	9.79541E-22
hsa-miR-133a	0.6874	306.0547	8.79842396	0
hsa-miR-449a	0.01	4.1407	8.69373087	1.23214E-18
hsa-miR-195-3p	0.01	2.2504	7.81403765	1.68736E-10
hsa-miR-449c-5p	0.01	1.9804	7.62964804	2.45229E-09
hsa-miR-133b	0.0625	9.9918	7.3207446	5.40583E-42
hsa-miR-204-5p	92.2431	2212.9552	4.58438944	0
hsa-miR-410	0.25	5.3109	4.40895636	9.13765E-19
hsa-miR-21-3p	4.1872	78.134	4.22189274	5.5898E-249
hsa-miR-10b-5p	55198.7661	628948.7014	3.51023443	0
hsa-miR-19a-3p	5.1871	51.3092	3.30621744	6.9257E-134
hsa-miR-4661-5p	0.125	1.1702	3.22675512	0.000345083
hsa-miR-199b-5p	1.5624	13.8625	3.1493517	1.00921E-35
hsa-let-7i-5p	248.6065	2045.6153	3.04059893	0
hsa-miR-144-3p	0.9999	8.1915	3.03427193	5.66076E-21
hsa-miR-486-3p	0.5	3.7807	2.91865338	4.6769E-10
hsa-miR-19b-3p	24.4357	181.8325	2.89554775	0
hsa-miR-941	1.9374	12.7823	2.7219537	1.49032E-28
hsa-miR-1468	1.4999	9.0916	2.59966789	9.12913E-20

The top upregulated and downregulated microRNAs (miRNAs) were based on the fold change value (log_2_). The fold change was the largest in the 40 upregulated miRNAs. The absolute value of the fold change (log_2_) was the largest in the 286 downregulated miRNAs.

a Standard expression values in NC samples;

b Standard expression values in MN samples

c Fold change formula: fold change = log_2_ (MN standard expression/NC standard expression).

MN, membranous nephropathy; NC, normal controls.

**Table IV:** Differential expression of novel miRNAs in the MN and NC groups.

Novel miRNA name	NC	MN	Fold change (log_2_)^a^	P-value
Downregulated				
novel_miR_82	1.1249	0.01	−6.81365294	8.92E-05
novel_miR_98	1.3749	0.01	−7.10318289	1.08E-05
novel_miR_89	1.6874	0.01	−7.3986582	7.75E-07
novel_miR_84	1.7499	0.01	−7.45112866	4.58E-07
Upregulated				
novel_miR_152	0.01	1.6203	7.34011714	8.70E-08
novel_miR_15	0.01	9.2717	9.85669008	1.01E-40

a Fold change formula: fold change = log_2_ (MN standard expression/NC standard expression).

miRNA, microRNA; MN, membranous nephropathy; NC, normal controls.

**Table V:** miRNA base edit comparison between the MN and NC groups (Top 10 ratio >1, 6 ratio = 1, top 6 ratio <1).

miRNA name	Count with base edit/total edit (MN)^a^	Count with base edit/total edit (NC)^b^	Ratio (MN)/ratio (NC)^c^
Ratio (MN)/ratio (NC) >1			
hsa-miR-1	25.59	0.48	53.20
hsa-miR-542-3p	16.55	0.40	41.80
hsa-miR-190a	9.09	0.28	32.80
hsa-miR-30b-5p	19.68	0.75	26.17
hsa-miR-146a-5p	22.82	1.26	18.13
hsa-miR-146b-5p	22.07	1.29	17.12
hsa-miR-128	23.23	1.98	11.73
hsa-miR-9-5p	24.48	2.17	11.28
hsa-miR-374b-5p	12.50	1.18	10.62
hsa-miR-28-5p	12.96	1.24	10.48
Ratio (MN)/ratio (NC) = 1			
hsa-miR-378b	100.00	99.55	1.00
hsa-miR-23c	100.00	99.67	1.00
hsa-miR-378f	100.00	99.72	1.00
hsa-miR-378h	100.00	99.76	1.00
hsa-miR-378i	100.00	99.99	1.00
hsa-miR-1304-3p	100.00	100.00	1.00
Ratio (MN)/ratio (NC) <1			
hsa-miR-378g	98.85	99.82	0.99
hsa-let-7e-5p	94.69	96.50	0.98
hsa-miR-19a-3p	78.09	81.60	0.96
hsa-miR-423-5p	0.87	0.92	0.95
hsa-miR-30e-3p	80.45	85.25	0.94
hsa-miR-421	10.64	11.48	0.93

a Percentage of miRNA base edits in MN samples.

b Percentage of miRNA base edits in NC samples.

Ratio (MN) and ratio (NC) are the values of a and b, respectively; c was used to make the comparison between a and b. If c >1, the base edit was more common in MN samples; if c = 1, the base edit was equivalent in MN and NC samples; if c <1, the base edit was more common in NC samples. miRNA, microRNA; MN, membranous nephropathy; NC, normal controls.

**Table VI:** Validation of miRNA expression profiles by qRT-PCR.

miRNA	Group	Ct_U6_	Ct_miRNA_	ΔCt = Ct_miRNA_ − Ct_U6_	Ct_(MN − CG)_ = ΔCt_MN_ − ΔCt_NC_	2^−ΔΔC^ a	MN/NC ratio^b^	log_2_ (MN/NC ratio)^c^
hsa-miR-7-5p	NC	10.88	21.61	10.73	0	1	0.03	−5.06
	MN	11.37	27.16	15.79	5.06	0.03		
hsa-miR-615-3p	NC	10.88	17.26	6.38	0	1	0.19	−2.40
	MN	11.37	20.15	8.78	2.40	0.19		
hsa-miR-577	NC	10.88	35.40	24.52	0	1	0.46	−1.12
	MM	11.37	37.01	25.64	1.12	0.46		
hsa-miR-98	NC	10.88	28.04	17.16	0	1	19.43	4.28
	MN	11.37	24.25	12.88	−4.28	19.43		
hsa-miR-375	NC	10.88	19.51	8.63	0	1	6.19	2.63
	MN	11.37	17.37	6.00	−2.63	6.19		

a 2^−ΔΔCt^ value, used to determine changes in expression levels in MN samples relative to NC samples. 2^−ΔΔCt^ = 2^−Ct(MN − NC)^.

b 2^−ΔΔCt^(MN)/2^−ΔΔCt^(NC).

c Equivalent to the fold change (log_2_) value provided in the microarray analysis of miRNA expression in the MN and NC groups.

If c <0, miRNA expression was downregulated; if c >0, miRNA expression was upregulated. miRNA, microRNA; MN, membranous nephropathy; NC, normal controls.
